# Renal Intercalated Cells: Alien Cells Inside Us?

**DOI:** 10.3390/biology14060607

**Published:** 2025-05-26

**Authors:** Miguel Luis Graciano

**Affiliations:** Nephrology Section, Department of Clinical Medicine, Universidade Federal Fluminense School of Medicine, Niteroi 24070-090, RJ, Brazil; mgraciano@id.uff.br

**Keywords:** evolutionary cell biology, metazoan origin, phenotypic plasticity, H^+^V-ATPase, cell energization

## Abstract

Mammalian renal intercalated cells are known for their role in acid secretion and maintaining acid–base balance. Herein, we discuss the theoretical reasons behind their development based on published data, focusing on the unique characteristics of renal intercalated cell biology that distinguish them from other mammalian cell types, while simultaneously attempting to explain the persistence of cells similar to intercalated cells throughout evolution. In addition, we traced these characteristics phylogenetically back to the simplest organisms. Intercalated cells have several functions and attributes. First, they contribute to kidney defense mechanisms in response to both infectious and non-infectious kidney damage. Second, intercalated cells are energized by V-ATPases in a manner similar to that of protozoa. Third, they possess T-antigens, which are commonly found in embryonic and cancer cells and which confer invasive abilities to these cells. Fourth, their plasticity enables the regeneration of other epithelial cells. These observations indicate that the origins of renal intercalated cells may be traceable back to amoeboid cells that originated from an evolutionary lineage including protists, or even to the last eukaryote common ancestor. The theoretical framework presented herein supports two predictions: first, that sponge amoebocytes possess membrane V-ATPase and are sensitive to bafilomycin, but not to ouabain; and second, that sponge amoebocytes—along with cells from diploblasts (such as Xenacoelomorpha), cnidarians, worms, fish and mollusk ionocytes, and the entire cell lineage containing V-ATPase, carbonic anhydrase, and anion exchangers (HCO_3_^−^/Cl^−^)—have innate immunity, cellular dedifferentiation, and regeneration capabilities.

## Simple Summary

Intercalated cells are specialized kidney cells responsible for excreting acid produced daily by our bodies. These cells utilize H^+^-ATPase, a proton pump, to carry out this function. This pump energizes the cells, creating an internal environment distinct from the external world. However, most animal cells depend on a different mechanism—the sodium–potassium pump—to achieve this function. Cells energized by H^+^-ATPase are commonly observed in simpler organisms, including certain animals and unicellular protozoa. These cells play a crucial role in specific functions such as cell invasiveness, immune response, and stem cell behavior, distinguishing them from more typical cells that rely on the sodium–potassium pump. By reviewing the biological literature and confirming the presence of H^+^-ATPase-harboring cells across multiple species, we propose that these cells can be traced back through evolutionary history. The coexistence of two distinct cell types within the same organ presents an evolutionary advantage, as it allows the organ to maintain normal function while a subset of cells focuses on defense and regeneration when necessary. Remarkably, the ability to develop and maintain two distinct cellular frames has been conserved throughout evolution, from the simplest organisms to mammals, highlighting its fundamental biological significance.

## 1. Introduction

Renal intercalated cells (ICs) are epithelial cells that reside in the collecting ducts of the kidney. They are involved in the regulation of blood pH, mainly through the secretion of H^+^ by an apical proton pump using active transport of the ion with an expenditure of energy. There are at least two types of renal ICs, denoted types A and B, which perform opposite functions. Type A cells secrete protons (H^+^) and absorb bicarbonate (HCO_3_^−^), while type B cells secrete HCO_3_^−^ and absorb H^+^ (there is also a non-A-, non-B-type intercalated cell that is not covered in this text). Type A cells are more common due to our typically acidifying diets

The two cell types display two different molecular arrangements. The more prevalent type A cell has a V-type H^+^ pump localized in the apical membrane, facing the interior of the renal tubule, where urine is being formed, which secretes acid directly into the urine, with energy expenditure. The H^+^ available for excretion is produced inside the cell through the hydration of CO_2_ under catalysis by carbonic anhydrase, ultimately forming HCO_3_^−^ and H^+^. The proton is secreted through the apical membrane by the H^+^-ATPase pump and the bicarbonate is absorbed into the systemic circulation, perfusing the renal interstitium in exchange for chloride by a counter transporter at the basolateral membrane (the anion HCO_3_^−^/Cl^−^ exchanger or SLC4A1). Type B renal ICs have a similar molecular construction, although with crucial anatomical and physiological differences. Like type A cells, type B cells contain carbonic anhydrase in their cytoplasm that generates H^+^ and HCO_3_^−^; however, the H^+^-ATPase pumps localize in these cells at the basolateral membrane, promoting proton movement in the opposite direction, from the forming urine to the blood and systemic circulation. Moreover, in contrast to A-type cells, the type B cell HCO_3_^−^/Cl^−^ anion exchanger is a distinct molecule called pendrin (or SCL26A4). In a noticeable example of phenotypic plasticity, type A and B ICs switch into one another depending on their exposure to a basic or acidic environment [1].

In addition to their well-known physiological role in acid–base control, renal ICs exhibit a series of characteristics that extend beyond typical mammalian or primitive cell behavior. Intrigued by this uniqueness, we embarked on an investigation based on the published literature in developmental biology, comparative physiology, and evolutionary cell and molecular biology. Our focus centered on unraveling the origin of these peculiar epithelial mammalian cells, which diverge significantly from conventional cells in terms of their behavior. Central to our inquiry was an examination of the physiology and evolution of cells that bear H^+^-V-type ATPases in their plasma membranes. This phenomenon is far less common than the presence of ubiquitous V-ATPases in intracellular organelles, such as lysosomes. Furthermore, there are mitochondrial ATP synthases that function as V-ATPases but in “reverse”, transporting H^+^ to synthesize ATP. Considering the unique nature of these cell types, our investigation also explored the molecular evolution of V-ATPases by examining their function within the plasma membrane of these specialized cells. Therefore, we hypothesize that renal intercalated cells possess the genetic and cellular machinery of a pluripotential cell, similar to protozoa, and thus belong to a long lineage of cells exhibiting similar characteristics.

## 2. B-Type Intercalated Cells Are Localized by Peanut Agglutinin

ICs are stained by lectin peanut agglutinin, which recognizes a set of sugars, including the Thomsen–Friedenreich antigen (T or TF antigen), composed of Galβ1-3GalNAcα1-R. TF antigens are rarely expressed in normal human cells; the cells in the collecting duct that are stained by peanut agglutinin, type B ICs, are an exception [2,3]. As we shall see later, this TF antigen confers invasiveness to embryonic and neoplastic cells.

## 3. Role of Intercalated Cells in Urinary Tract Infection and Urosepsis

ICs have the appearance of typical epithelial transport cells, which are vital in acid–base regulation, in addition to being the key cells defending the kidneys against ascending urinary bacterial infection.

Uropathogenic *Escherichia coli* (UPEC) is the main pathogen causing ascending urinary infection. It has been shown that α-ICs facing urinary tract infection (UTI) phagocytose *E. coli* increase H^+^-ATPase expression and acidify phagolysosomes [4]. These cells, when challenged with UPEC, produce RNAse 4 and human beta defensin 1, as well as the antimicrobial peptides cathelicidin, lipocalin 2 (NGAL), and calgranulin [5]. The pro-inflammatory cytokine IL-18 in the kidney is almost exclusively expressed in collecting duct ICs (both A and B). It is yet to be confirmed that IL-18 elicits an epithelial response to UTIs; nonetheless, they are pathogenic in lipopolysaccharide-induced acute kidney injury [6].

It should be noted that H^+^-ATPase works together with carbonic anhydrase to fulfil the acid production (from CO_2_ + H_2_O) and transportation to the renal cortical collecting tubule lumen. Interestingly, carbonic anhydrase II (CAII)-knockout mice showed increased susceptibility to pyelonephritis, both in the whole-animal model and in the knockout being selectively restricted to the kidneys [7,8].

## 4. Role of Intercalated Cells in Non-Infectious Kidney Damage

Renal ICs, in addition to being immune cells combating infection in the kidney, are also involved in the pathophysiology of renal damage not associated directly with renal infection. Acute kidney injury (AKI) is defined as an abrupt and usually reversible decline in the glomerular filtration rate. Serum creatinine levels and urinary output—despite being the cornerstones of AKI diagnosis in clinical medicine—are late-stage markers of the disease, indicating whole-organ dysfunction rather than the subtle cell damage that occurs earlier. Therefore, newer biomarkers have been identified that might support preventive treatment of this frequently lethal disease, such as NGAL and IL-18. These biomarkers were proposed to predict AKI clinical outcomes (dialysis or death) [9,10].

The most frequent mechanism of AKI, systemic inflammation, whether sterile or septic, is mediated by innate immunity-induced inflammation. Two components of the innate immune response, NGAL and IL-18, might be involved in the defense against UTIs. More importantly, CD ICs seem to be the source of both molecules in the kidney [11,12]. IL-18 participates in the innate immune response in acute renal injury sepsis early in the organ response to the insult, even prior to the clinical diagnosis of the disease [6]. Lipocalin 2 is an important contributor to the innate defense response given its bacteriostatic nature of depriving bacteria of available iron. As such, agents mimicking NGAL’s actions on iron metabolism have been investigated as anti-infective agents for human UTIs [13].

In acute kidney injury, the tubule segments more likely to suffer from ischemia have high metabolic rates and oxygen consumption, such as the pars recta of the proximal tubule (S3) and the thick ascending limb of Henle. Interestingly, however, renal collecting duct cells that already live in oxygen scarcity have been found to be more likely to survive ischemic events [14]. There has been much discussion about whether the main model of AKI, in which renal arteries are clamped to block blood flow, causing acute tubular necrosis, is the best model to describe human AKI. A recent publication with more refined histological techniques (electronic microscopy and immunohistochemistry) described the renal lesions from 41 patients who later died of sepsis. It was shown that cell death, although not frequent (5% of cells) but prevalent (44% of patients), and focal acute tubular injury were present in a majority of the cases (77%) [15]. This injury, however, did not translate to the severe degree of renal dysfunction observed in such cases. The fact that there is rarely a complete cessation of blood flow in human AKI may explain the discrepancy between clinical and experimental AKI. It is important to emphasize that sepsis, not hemorrhage, is currently the main cause of AKI in human patients [16]. Similar tubular damage does occur in catastrophic hemorrhage, however, which might be better represented by ischemia–reperfusion injury (IRI) models due to the arterial clamping. The abovementioned study also reported medullary tubular epithelial cell sloughing (97%) and, rather interestingly, the presence of phosphate crystals in 50% of the cases. This may indicate damage to ICs, leading to calcium phosphate precipitates in an alkaline environment.

However, the IRI experimental model provides informative elements, e.g., permitting the investigation of very small molecular details such as the integrity of tight junctions vital to the functional polarity of epithelia. The loss of tight junctions was observed in the collecting ducts, particularly the tight junctions of ICs, and mainly those that were detached [17].

In fact, in a model of kidney ischemia–reperfusion injury, the damage-associated molecular pattern molecule UDP-glucose bound to the purinergic receptor P2Y14 on the apical side of α-ICs, initiating an inflammatory response [18,19]. Moreover, the inhibition of the P2Y14 signal abrogated the same inflammatory response [20]. We should also mention that urine UDP-levels correlated with the incidence of AKI in ICUs [20]. It is thus conceivable that regardless of the site of injury to the renal tubule, the ICs from the collecting ducts are responsible for mounting and amplifying the inflammation that characterizes damage.

## 5. Kidney Epithelial Regeneration After Damage

Data have been found supporting the idea that renal ICs can repopulate damaged tubular epithelium in renal obstruction [21,22,23]. When this occurs, there is an early increase in ICs, followed by the proliferation of principal cells repopulating the tubule. Importantly, B-type ICs can effectively differentiate between both A-type ICs and principal cells (PCs), the latter of which are the main cellular type in this nephron segment [24]. It is particularly relevant that the stem cell marker CD117 (c-kit) gene expression has been observed in single-cell preparation of intercalated cells, underscoring the capability of these cells to promote tubular regeneration [25]. Furthermore, ICs can breach and invade the basal membrane, forming a collar of dedifferentiated cells [21], an activity compatible with the presence of the invasiveness marker, the TF antigen. Another point of convergence between ICs and tissue-invading cells is that amoeboid cancer-invading cells are triggered by hypoxia-induced factor (HIF) [26]. Coincidentally, ICs are also activated by HIF in a model of collecting duct hypertrophy employing the SGLT2 inhibitor empagliflozin [27]. SGLT2i inhibits sodium and glucose reabsorption in the proximal tubules. The surplus sodium load is then reabsorbed in more distal renal segments, rendering the environment around the collecting ducts an even more critical area of oxygen demand.

Retinoid acid or vitamin A is a known early hormone activator of embryonic kidney development of the ureteric bud that forms the CD and the metanephric mesoderm [28,29]. ICs from mice challenged with transurethral inoculation of UPEC showed downregulation of the retinoic receptor Rxrα. ICs showed high titers of Foxi1, the transcription factor related to IC cell differentiation, and a series of inflammatory mediators [30]. Renal malformation was shown to occur with vitamin A deficiency [31,32]. RA expression persists until adulthood in the collecting duct, both in principal cells (PCs) as well as ICs [33].

Calcium phosphate deposits in human kidney tissue with sepsis and with a diet poor in vitamin A. Curiously, rats fed a low-vitamin A diet were prone to renal infection and urolithiasis, which is compatible with collecting duct defects in infection defense and acid excretion, particularly in the case of phosphate stones, typical of alkaline urine [34]. The same was observed in sepsis [15]. Therefore, ICs appear to participate in the induction phase of innate immunity in AKI as well as in its recovery phase.

## 6. Renal Intercalated Cells Are Energized in a Peculiar Way

Following Skou and Hodgkin and Huxley’s [35,36] seminal works, it was realized that Na^+^, K^+^-ATPase resident in the plasma membrane of animal cells is the fundamental enzyme pump that creates and maintains a different environment inside and outside the cell, with the interior electrically negative, poor in sodium, and high in potassium; thus, a paradigm was created. Accordingly, all other transportation across cell membranes is secondary to the electrochemical potential created by the action of enzymes with an expenditure of energy. Epithelial cells have a special molecular arrangement in which the Na^+^, K^+^-ATPase is confined only to the basolateral membrane facing the interstitium of the organs they dwell in, contacting connective tissue and systemic capillaries outside the tubules. Therefore, they are directly connected to the extracellular fluid and its composition (low-potassium, high-sodium environment). Meanwhile, the other side of the cell, the apical side, “sees” the outside world, with urine formed in the interior of renal tubules, food digested within the bowels, and similar scenarios. The paradigm therefore created is known as the Ussing model after Hans Ussing’s experiments on frog skin ion transport [37].

**Figure 1:**
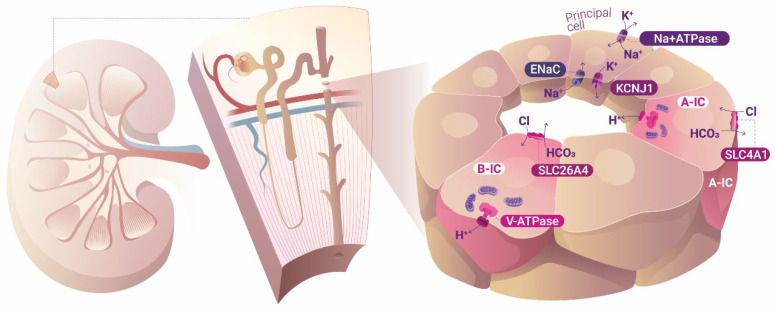
Ion transport in the cortical collecting duct. Intercalated cells are energized by V-type H^+^-ATPase and can interconvert between A- and B-type ICs according to the acid–base status of the organism. Principal cells, on the other hand, are energized by the ubiquitous mammalian Na^+^ and K^+^-ATPase. ENaC, epithelial sodium channel; A-IC, A-type intercalated cell; B-IC, B-type intercalated cell.

This scenario is observed in the main cell type of the collecting duct (CD), the PCs. In the specific case of the PC, the most common sodium transporter dwelling at the apical membrane is the epithelial sodium channel (ENaC), through which Na+ enters the cell without any accompanying ion or substance, driven solely by the chemiosmotic force generated by the operation of Na^+^, K^+^-ATPase in the basolateral membrane. Therefore, a negatively charged intraluminal urinary tubule is created in comparison to the basolateral parenchymal interstitial space, which in turn helps the transport of positive cations, such as K^+^, from the cell into the tubule lumen. In Figure 1, this cellular arrangement is schematically represented in the principal cells of the collecting duct (CD). However, in contrast to most animal cells, CD ICs are energized by V-type H^+^-ATPase (in this text, we employ V-type H^+^-ATPase, V-ATPase, or H^+^-ATPase unless otherwise specified; P-ATPase is always referred to as such), and not Na^+^, K^+^-ATPase, which is also applicable to protozoa and archaea. Accordingly, bafilomycin (a V-ATPase inhibitor) depolarized ICs and caused cell swelling, but had no effect on PCs, whereas ouabain (a Na^+^, K^+^-ATPase inhibitor) caused the opposite effect [38]. Figure 1 describes the ion transport that takes place in both PCs and ICs. Long after the influential Ussing model established that plasmalemma Na^+^, K^+^-ATPase is the primary cell energizer and that all other transport phenomena occurring across the cell membrane is secondary to the initial movements of Na^+^ and K^+^ made by this energizer, Wieczorek proposed a second mechanism of cell energization initiated by the primary transport of H^+^ by V-ATPase, which also creates opportunities for secondary transport to take place [39]. This concept was conceivable only after the discovery of the presence of V-ATPase in the plasma membranes of animal epithelial cells, and not only inside the cells themselves [39].

In light of the above, at least two types of such cell-energizing enzymes have been identified. (1) The first type are enzymes employing a phosphate intermediate during operation, and are thus known as P-ATPases; this is the group to which the Na^+^, K^+^-ATPase, and the H^+^/K^+^-ATPase acid secretor in the stomach belong. P-type H^+^-ATPase, which is common in plants, algae, and fungi, energizes cells in this manner. Accordingly, vascular plants that are evolutionarily more complex are devoid of Na^+^, K^+^-ATPase in their plasma membranes [40]. Humans, vertebrates, and invertebrates usually do not possess P-type H^+^-ATPase to energize their cells. The load of the cell energy is produced in the more common P-type ATPase, the ubiquitous and pervasive Na^+^, K^+^-ATPase. (2) The other known types of H^+^-ATPases are the V-type, usually found in intracellular vesicles or vacuoles (named “V”-ATPases) and lysosomes, and on the plasma membrane of certain special types of cells; while F-ATPase is encountered in mitochondrial crista and plastids. Both V- and F-type H^+^-ATPases are rotatory molecular machines that may operate in two different manners: the first type cleaves ATP and uses the released energy from this reaction to transfer H^+^, and the second type transfers H^+^ and consequently stores electrical energy inside ATP molecules. F-ATPase is also encountered in plasma membranes of bacteria and can operate in both directions, either synthesizing ATP or transporting H^+^. In animals, F-ATPases only synthesize ATP inside mitochondria (they are also known as ATP synthases), while V-ATPases pump H^+^ into endosomes. There is also another type of V-ATPase, called A/V-ATPase or A-ATPase, that is found in archaea, the third domain of life (the other two are bacteria and eukaryotes). In sum, P-ATPases, whether H^+^ or Na^+^, K^+^, energize cells of plants and animals as the rule. The same is true for F-ATPases energizing prokaryotes and V-type ATPase energizing vesicles and endosomes. The unusual scenario is the V-type ATPase energizing the plasma membrane of animals, as we verified in renal ICs.

The renal collecting duct is a mosaic epithelium in which the predominant type of cell is PCs, with ICs representing a minority. The voltage across this renal epithelial segment is determined by the more prevalent principal cells employing the universal mode of energizing cells in the animal world, through the active pumping of Na^+^, K^+^-ATPase in the basolateral membrane of PCs. The transepithelial voltage in the cortical CD is usually 10–50 mV negative inside the epithelia, while the energizing V-type H^+^-ATPases can generate voltages on the order of 100–200 mV positive inside epithelial tubules. Consequently, this negative voltage inside the CD could never be produced by the pumping of positive protons. Therefore, it is conceivable that proton secretion is prioritized in this segment, rather than positive voltage creation.

Other examples from the animal domain that employ V-ATPase as energizers are organisms living in fresh water in a very low salt environment that need to actively absorb salt to maintain the high-sodium profile of the extracellular fluid. Some examples include frogs living in fresh ponds, clams, and freshwater fish. The sodium extrusion mechanisms of sea-water fish have long been known; however, the salt reabsorption mechanisms of freshwater fish are still unclear. An immediate and simple explanation for this disparity is that it could be caused by saltwater sodium extrusion systems relying on Na^+^, K^+^-ATPases residing on the basolateral membrane of the fish gill, with the CFTR (cystic fibrosis transmembrane rectifier, named after the human disease) extruding chloride through the apical membrane to sea water, followed by passive disposal of sodium, corresponding to the well-established Ussing model [41]. The problem with freshwater animals derives from the fact that they need to absorb sodium from a low concentration in a steep gradient without loading the interior of the epithelial cell with an alkaline ion. The answer may lie in the energization of the gills of freshwater animals by the V-type ATPase, as it has been recently demonstrated that the long-sought apical sodium transport coupled with the primary action of a V-ATPase exists in the gills of freshwater fish [42].

In light of the above, we inferred that the energization of animal cells by the V-type H^+^-ATPase is an exception. However, some human cell types—such as macrophages, osteoclasts, cochlear cells, and epididymal cells—operate in this manner. In the following section, the similarities and differences between these cells and renal ICs are discussed.

## 7. Cochlea and the Endolymphatic Sac

A surprising aspect of cochlea physiology is the composition of the endolymph inside the scala media, consisting of a high positive potential of +80 mV relative to the perilymph in the other two coiled tubes (scala vestibular and tympani) that compose the cochlea spiral, snail-shaped hearing organ. Additionally, the endolymph is similar in composition to the intracellular fluid, with a potassium concentration of 150 mEq/L and a very low sodium concentration, i.e., 1 mEq/L [43]. This transepithelial voltage is much higher than what is found in human kidney segments where the electric potential difference is the highest (provided by a mechanism involving Na^+^, K^+^-ATPase energization): about +50 mV in the thick ascending limb of Henle and <−50 mV in the collecting duct.

The peculiar composition of the cochlear endolymph has been ascribed to a “potassium pump” or complicated models involving energization by Na^+^, K^+^-ATPase according to the traditional Ussing model of epithelial ion transport. The situation here is very similar to what has been observed in *Manduca sexta* and the mosquito midgut, which has a highly positive electrical potential difference of about +100 mV and a potassium concentration like that of the intracellular environment [44]. The “potassium pump” in the insect cases was explained by the coupling of a H^+^-ATPase to a H^+^/K^+^ counter-transporter harboring a 1:2 stoichiometry. Similarly, the cochlear endolymph composition is equally well explained by the combination of a V-ATPase and H^+^/K^+^ antiporter [45]. Importantly, it has been observed in cochlear cells that V-ATPase, CA, and the Cl^−^/HCO_3_^−^ exchanger, like ICs, are necessary for the production and secretion of H^+^ [46]. It is universally believed that the stria vascularis is the ear organ that produces the peculiar composition of the cochlear endolymph. The strongest expression of V-ATPase in the cochlea is precisely in the stria vascularis, which also contains CA and Cl^−^/HCO_3_^−^ exchangers [46,47]. There is another structure in the ear which looks more like the renal CD, the endolymphatic sac (ES). The ES drains the endolymph to the cerebrospinal fluid. Its epithelial layer is composed of cells containing V-ATPase, CA, AE, and pendrin, very similar to the renal CD. Moreover, the ES’s pH is acidic, despite the fact it communicates with the neutral cochlear endolymph. The role of V-ATPase both in the cochlea (EP generation and potassium secretion) and endolymph (pH regulation) has been emphasized by physiological studies demonstrating that the V-ATPase inhibitor bafilomycin abrogates both functions, while the Na^+^, K^+^-ATPase inhibitor ouabain has no effect [48,49].

Interestingly, the ES may clear cellular debris in the endolymph and provide immune capacity to fight infections, such as meningitis or otitis [50,51]. The cell composition of the ES includes a major component referred to as ribosome-rich cells and a minor population of mitochondria-rich cells. These mitochondria-rich cells contain carbonic anhydrase, V-ATPase, and AE (SLC4A2) on the basolateral membrane [46] and pendrin on the apical membrane [52].

## 8. Epididymal Cells

Acidification of semen keeps sperm inactive until ejaculation, when they come into contact with prostate fluid [53,54]. Like the anatomy and function of the CD, the epididymis also comprises two main types of cells, resembling the collecting ducts of the kidney, the principal cells and the mitochondrion-rich (MR) cells that possess V-ATPase in their apical membrane [39]. Moreover, they contain CA in their cytoplasm and pendrin as Cl^−^/HCO_3_^−^ AE in the basolateral membrane [55]. In another resemblance to ICs, epididymis MR cells were stained by peanut agglutinin [56]. The similarities do not stop here, for MR cells can mount an innate response against pathogens in the male reproductive tract [57].

## 9. Macrophages

Macrophages are commonly described as the per se phagocytizing cell, engulfing foreign biological materials as well as decomposing host cells, thereby acidifying endosomal vesicles called phagosomes through the action of V-ATP to help digest the ingested material, with proteolytic enzymes “secreted” into the vacuoles. Given that this process is old, it is unclear which evolved first: the eukaryotic cells or phagocytosis [58,59]. Furthermore, macrophages also contain V-ATPase in the plasma membrane and are energized by the enzyme, in addition to binding to and being histochemically detected by peanut agglutinin [60,61]. Accordingly, the unexpected similar features of macrophages and ICs are participation in innate immunity, phagocytosis, the presence of H^+^-ATPase in the plasma membrane, and staining by peanut agglutinin. Moreover, the inducible isoform of nitric oxide synthase, iNOS, typical of macrophages responding to inflammation, was detected in the apical region of renal ICs [62].

The macrophage cytoplasm pH is maintained by V-type H^+^-ATPase and is inhibited by bafilomycin [63]. It was found that macrophages possess V-type H^+^-ATPase on the plasma membrane that is inhibited by the enzyme-specific antagonist bafilomycin [64,65]. Monocytes and macrophages react with PNA and display a range of staining intensities, with the intensity being greater in more immature cells [60]. Most interestingly, T antigen is found on embryonic macrophages [61], a cell type that can penetrate tissues in a manner akin to metastatic cancer [66,67]. The T antigen or TF antigen is an oncogene that is expressed in tumor cells and invasive embryonic cells. The glycosylated form of T-antigens bears the NAC-Gal epitope, recognized by lectin peanut agglutinin. The staining of cells with PNA, therefore, although not specific, is commonly observed in cancer cells. Interestingly, no type of human renal cancer cells can be identified by PNA staining except for renal chromophobe cancer, which derives from renal ICs [68]. It is likely that, in this case, only the cell origin of the neoplasm—and not neoplastic behavior or abnormal invasiveness—is demonstrated. The simplest form of the antigen is GalNAcα1-Ser/Thr (serine or threonine), also known as the Tn antigen (from T nouveau, for they were discovered after the Thomsen–Friedenreich antigen). T antigen is formed after the addition of Galβ1-3 to the Tn antigen, forming the Galβ1-3GalNAcα1-Ser/Thr structure. Both are usually hidden in most cells by sialic acid or other molecules. However, cancer cells have these antigens exposed, which is linked to invasiveness, metastasis, cell adhesion, and angiogenesis, and their presence usually carries a poor prognosis. A few normal cells have exposed T antigens that are detected by the plant lectin peanut agglutinin: type B renal ICs, osteoclasts, macrophages, and epididymal cells; the same group of cells that are energized by V-type H^+^-ATPase [69].

The following question arises as a result of the above: why do B-type renal ICs possessing the T antigen always express their mature form, and why does this not cause harm? This could be because B-type renal ICs in their quiescent state do not exhibit macrophage-like lineage behavior. This type of behavior typically occurs when there is damage to the renal epithelium. Accordingly, another question is raised: why is an embryologically important mechanism still important in an adult renal epithelial cell? This could be because, when needed, ICs can digest and cross the epithelial basement membrane, thus becoming invasive [21].

Phagocytic cells are very old in eukaryotic history, at least 1.2 to 1.6 Gy [59], with more evolved forms of phagocytosis consistent with the end of the Mesoproterozoic Era [70]. These more complex forms of phagocytosis are found in Protozoa phylogenetically more related to Metazoa, such as Amoebozoa [71]. It is of utmost importance here to investigate how the proximal phylogenetic developments of the earlier Metazoa happened, particularly from Amoebozoa and coanoflagellates to the early animals (Porifera), and how this might have evolved to be represented in current animals, including humans.

## 10. Osteoclasts

Osteoclasts are cells that are constantly helping to remodel bone through reabsorption. They are a syncytium of resident macrophages in the bone residing at the reabsorbing surface. Osteoclasts harbor H^+^-ATPase in their plasma membrane that secretes acid and enzymes to destroy both the mineral and organic components of bone. Osteoclasts possess the complete set of transporters to secrete acid, apical H^+^-ATPase, intracellular CA, and basolateral Cl^−^/HCO_3_^−^ AE. They are also involved in bone innate immunity, including osteomyelitis [72] and periodontitis [73], and are stained by peanut agglutinin [74]. Accordingly, osteoclasts have roles that are not related to bone reabsorption, but rather to immune functions typical of macrophages, such as disposal of apoptotic cells [75,76], antigen presentation [77], cytokine production (also known as clastokines in the case of osteoclasts) [78], phagocytosis, and T-cell activation [79].

## 11. Protists and the Phylogeny of Macrophages

It should be noted that the similarities between ICs and macrophages may reveal that the latter is an ancestor line of the former, because macrophages are primordial cells that can be observed in every multicellular type of life. Moreover, because eukaryotic life is intermingled with the acquisition of mitochondria by ancient pre-eukaryotic forms of life through phagocytosis, even in its primitive form, the original forms of macrophages can often be confused with eukaryote life itself [58,59]. In this regard, Bajgar and Krejčová retraced the pathways of macrophage phylogeny in animals and, “while investigating the origin of macrophage-like cells in the animal phyla, [they] realized that macrophage-like amoebocytes are present in virtually all multicellular animals”. Therefore, they investigated macrophage-like cell evolution in prokaryotes, starting with predatory unicellular organisms that basically employed phagocytosis to feed, to social unicellular amoebas, and then to the simplest form of multicellular animal life, the choanoflagellate, the simplest animals Porifera, and finally, a full-blossomed animal, the fruit fly, *Drosophila melanogaster*, tracing a phylogenetic line among them [80].

**Figure 2:**
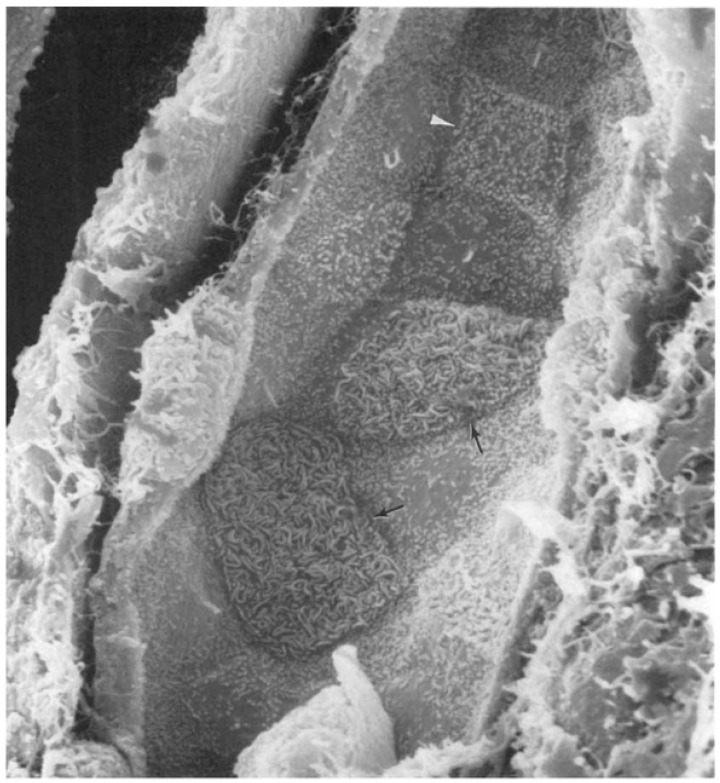
Scanning electron micrograph illustrating the luminal surface of a rat cortical collecting duct. The principal cells possess a single cilium. Type A intercalated cells (arrows) present with a large luminal surface covered with microplicae, while type B (arrowhead) cells have a more angular outline and a surface covered with small microvilli. Reprinted with permission from Madson et al. [90].

The phylogeny of the animal kingdom (Metazoa) that is currently most accepted claims that they derived from a unicellular group called Amorphea, which comprises the Amoebozoa and the Opistokonta (plus Apsomonada and Breviates), a group that in turn gave rise to fungi and animals as well as the closest unicellular organisms to animals, the choanoflagellate Protozoa group [81,82]. This protozoon (choanoflagellate) is particularly interesting because it is morphologically essentially identical to the choanocyte, the flagellated cell that makes up the body of sponges together with the amebocyte [83]. DNA data confirm this close relationship [84]. Because amoebocytes, also known as archeocytes, can by themselves generate an entire sponge, they are considered to be the primordial cell type of all multicellular animals [85]. Porifera choanocytes are in many aspects indistinguishable from the closest unicellular animal, the choanoflagellate [86]. Independent of their evolutionary order of appearance, feeding and sentinel roles observed in sponges have also been seen in the unicellular *Naegleria gruberi*, which can switch from an amoeboid to flagellate form [87]. Such a phenomenon is repeated in Euglena [88]. This distinction was preserved throughout evolution until humans, with the less differentiated phagocytizing cells receiving different names, amoebocytes, hemocytes, or macrophages, depending on the species they belong to. The later addition of choanoflagellates to this list strengthened this proposition [89]. We are extending this concept to include in the amoeboid lineage the renal epithelial ICs. It may sound curious that principal cells in the collecting tubules possess a cilium while their neighbors’ ICs have none, as depicted in Figure 2. While the cilia and flagellum are different structures, the possible meaning of this parallel comparison, i.e., the similarities between choanocytes and amoebocytes in sponges, “did not escape our notice”. The Naegleria study used a very robust approach to demonstrate that the interconversion between the amoeboid and flagellated form was determined by two large sets of genes that could be viewed more comprehensively as describing what we will define later as a “megalomodulon”.

We are theorizing here that there is a long line of evolution preserving these two types of cells, choanocytes or feeding cells, starting from protists, and maybe even the last eukaryotic common ancestor (LECA), spreading through aggregating social amoeba, “pre-animal” choanoflagellates, simple cnidarians, and Porifera, all the way to mammals, here represented by ICs and principal cells in the renal tubules of the CD. Of course, such an arrangement must pass the test in the embryonic or developmental period at every generation, when new sets or modules of terminal transcription factors are turned on at the proper time to confer differential labor division to every new type of cell from this long line of succession.

There are similarities among primitive amoeboid cells, macrophages, and renal ICs in terms of their feeding, immunity, and damage repair mechanisms. Furthermore, Protozoan and Amoebozoan cells share other features with Metazoan cells such as mammalian renal ICs, fish cells, amphibian cells, crustacean cells, and arthropod mitochondria-rich cells. The first feature is much closer to the function of the kidney, osmoregulation. In Protozoa, osmoregulation is performed by the contractile vacuolar complex (CVC). Without a cell wall, like bacteria and plants, to contain osmotic pressure effects inside the boundaries of the cell, protozoa depend on constant fluid extrusion by CVCs. Accordingly, in Paramecium, a free-living, nonpathogenic protozoan, there is more V-ATPase on its CVC membrane than on the phagosome, indicating that it has greater energy needs for osmoregulation than for feeding. However, despite the greater presence of H^+^-ATPases in CVCs, they are not acidified; in other words, H^+^-ATPases provide energy for the extrusion of fluid, but not for the acidification of CVCs [91]. Notably, the CVC membrane is a high-voltage (−80 mV) membrane; this was dissipated using the V-type H^+^-ATPase inhibitor concanamycin B, decreasing the voltage and the fluid extrusion by half in experimentation with Paramecium [92]. Amoebas are phylogenetically closer to animals. The pathogenic Entamoeba histolytica shares the same mechanisms for water and ion disposal and osmotic regulation and has V-ATPases in its plasma membrane [93]. The social amoebas capable of forming colonies, *Dictyostelium discoideum*, also use this mechanism [94]. Assuredly, the same mechanism is conserved in over a billion years of evolution [95]. To the best of our knowledge, V-ATPase has not yet been identified in Porifera; nevertheless, these simple animals also rely on CVCs to control osmolality [96]. Accordingly, cnidarians in a symbiotic relationship provide CO_2_ for algae photosynthesis through plasmalemmal V-ATPases. Therefore, animals that are more evolved than Porifera and less evolved than mollusks possess V-ATPase in their plasma membrane [97].

There is a gray zone considering osmoregulation of the so-called non-nephrozoan animals, i.e., those without nephrons or nephridia, which is a rudimentary nephron. At least one type of tubular excretory system is present from Bilateria (or triploblasts) to higher vertebrates. This gray zone encompasses the diploblasts, Porifera, Placozoa, Cnidaria, and Ctenophora. One may include in this group the choanoflagellate as the closest unicellular organism to the Metazoa. There is a dearth of information about osmotic regulation over these animals [98,99]. The major texts dealing with the matter refer to a sole paper from 1978 [97], reporting that CVCs also occur in sponge. This led some to suggest the disappearance of V-ATPases from the plasma membrane so common in protozoa, and its later evolutionary resurrection in triploblasts, such as mollusks. During this period, the H^+^-ATPases absconded inside the cells and were only present in phagosomes to provide acid for digestion. As discussed above, in a study on innate immunity evolution [80], it was shown that amoebocytes are pervasive in animal life, from amoebas themselves to mammals and primates. We have seen that sponges also bear CVCs. Since Darwin, there has been much discussion on missing links in evolution. One such living example is represented by the phylum Xenacoelomorpha, non-nephrozoan Bilateria animals. They form a boundary group of animals between diplo- and triploblasts which were recently shown to have anion pumps and transporters, such as SLC4, SLC12, SLC26, aquaporins, CA, and V-ATPase and Na^+^, K^+^-ATPase akin to kidneys and nephridia in their guts [100,101]. The same happened in the less evolved diploblasts and the non-bilaterian metazoan, the Cnidaria *N. vectensis* [101]. The transporters are not yet arranged in a coherent excretion organ. One may conceive two periods of great evolutionary leaps. The first occurred during the microbial world when macromolecules developed, and the second occurred during metazoan life when newer spatial configurations rendered new functions to older molecules depending on their anatomical positioning in different organs. Initially, there was an inchoate disposition of “excretory” molecules, then nephridia, and finally, kidneys. We previously introduced the concept of a “megalomodulon” to describe exactly this idea of a very large number of preexisting genes and transcription factors reshuffled at will during the evolution of complex organs in Metazoa. Megalomodulon comes from an extension of the term modulon, which refers to a set of regulons controlled by different regulatory transcription factors. A regulon is understood as a set of genes controlled by one specific regulatory gene; the set V-ATPase, CA, AE in ICs has been already referred to as a regulon [102]. As mentioned before, both V-ATPase and CA are involved in immune mechanisms preventing urinary tract infection [7,8].

## 12. Discussion

Based on the above theoretical reasoning, the following two predictions can be considered reasonable and straightforward. First, sponge amoebocytes contain membrane V-ATPase and are sensitive to bafilomycin, but not to ouabain. This hypothesis can be tested through DNA genome searches, employing bioinformatics resources to prove that the genes are present in sponge, as well as by immunohistochemistry, using primary antibodies directed against putative V-ATPase epitopes. Moreover, functional studies can be conducted to measure the response of the resting electrical potential of these cells to the use of specific ATPase blockers, such as bafilomycin or ouabain. Second, sponge amoebocytes, along with the entire lineage of V-ATPase powered cells, are present in xenoacelomorphs, cnidarians, and worms in general. In addition to plasma membrane V-ATPase, we predict that these cells contain carbonic anhydrase and an anion exchanger (HCO_3_^−^/Cl^−^), as these three proteins are necessary for proton generation and the transfer of protons from inside to outside the cells. Carbonic anhydrase enzymatic activity is a necessary step to produce protons from CO_2_ and water, generating carbonic acid which, in turn, releases proton ions to be transported by H^+^-ATPase. All the cell types mentioned in this paper contain CA that works in this way; some examples include macrophages, osteoclasts, fish gill cells, and mosquito gut cells. Particularly, sponge cells that make carbonate must function by a combination of CA and H^+^-ATPase. In the case of simpler animals like corals, this combination is also necessary, even if corals must rely on symbiosis to have the appropriate set of enzymes [91].

We also predict that the same cells possess innate immunity (in the form of Toll-like receptors and antimicrobial peptides) and molecular machinery conferring dedifferentiation and invasiveness abilities (such as the presence of a molecule similar to the Thomsen–Friedenreich antigen) and regeneration (possibly through pathways such as Wnt and TGF-beta, which have already been shown to be present in sponges [103]). Indeed, these insights could eventually lead to the development of new medications. For example, in acute kidney injury, a deeper understanding of the regeneration process may facilitate the creation of methods to promote tubular cell replenishment without scar formation.

These assumptions may eventually have implications in several medical fields, including regenerative medicine, AKI, cancer biology, and medical treatments based on a cellular organization different from that of typical mammalian cells. If one of these two basic cell plans, such as the one employing H^+^-ATPase, is the cell type more involved in regeneration and repair, one might concentrate research efforts on the particular biology of this cell type. One such example is the case of acute renal failure mentioned above. Additionally, if these cell types are key players in cancer invasion, knowing key points of their biology could lead to the discovery of drugs that might help contain cancer invasion. In the case of biomedical engineering, one may consider the design of artificial kidneys, including the current research in organoids. Knowledge of the biology of cells powered by H^+^-ATPase could provide information on the operation of developmental modules that can lead to the formation of specific structures and the construction of organoids.

The hypothesis presented herein states that there are two basic cellular plans in animals, including mammals: one consists of cells energized by Na^+^, K^+^-ATPase, and the other is powered by V-type H^+^-ATPase. Cells in the second subset, i.e., those energized by H^+^-ATPase, are particularly important, as they play a role in development and regeneration, as well as in neoplastic behavior. The implications of this hypothesis are immediate, as this might enable targeting the biology of specific cell behaviors in fields such as the treatment of neoplasia or epithelial regeneration. Moreover, this insight could change the dominant approaches in biomedical engineering, including in the construction of organoids. Focusing research on the biology of this subset of cells, including the understanding of their transcription and growth factors networks, could provide new therapeutic and diagnostic opportunities that would otherwise be missed if the body’s cells are all considered to share the same general plan.

Evolutionary cell biology is another important factor to consider. If the reasoning presented here holds true, it becomes possible to trace not only molecules, but an entire cell plan phylogenetically. From the perspective of organismal biology, this represents a turning point. We might suggest that molecules evolved vastly over the billions of years of microbial life, while cells, as a complete set of molecules and genes, evolved more recently, and at a faster pace.

## 13. Conclusions

In conclusion, we discussed theoretical reasons, based on published data, to believe that renal ICs are directly derived from amoeboid cells that originated from an old evolutionary line that includes many unicellular eukaryotes (protists), and maybe even the LECA or “urmetazoan”. Some arguments constructed to substantiate this proposition include the observation that ICs, like protozoa, are energized by a V-type H^+^-ATPase instead of the common metazoa Na^+^, K^+^-ATPase. Furthermore, ICs share some common innate immune functions with amoeboid-like cells: phagocytosis, cytokine and antimicrobial peptide secretion, TLR and NFκB signaling, antigen recognition, and the production of iNOS under stimulation. Moreover, the expression of the developmental/oncogene product T antigen confers invasiveness to B-type ICs. In addition, ICs possess stem cell-like behavior, as they can repopulate the tubular epithelia after acute kidney injury; these are characteristics of phylogenetically old or embryological cells. The fact that these cells have all these cellular characteristics in common suggest they share a common primitive origin. Such features are also shared by other cell types in the human body, macrophages and their syncytial version, the osteoclasts, epididymal cells, and endolymphatic sac cells. These same aspects are also shared in different animals by the “mitochondrion-rich cells” (MRCs) or ionocytes, and chloride cells in fish and crab gills, shark rectal glands, frog skin, insect midgut, and Malpighi tubules [98]. Here, we also presented reasons to believe that simple animals such as sponges or cnidarians also share the same heritage.

## Data Availability

Data are contained within the article.
